# NEIGHBORHOOD ENVIRONMENTAL CORRELATES OF FALLS AMONG OLDER ADULTS – IMPLICATIONS FOR FALLS PREVENTION PROGRAMS

**DOI:** 10.1093/geroni/igae098.3871

**Published:** 2024-12-31

**Authors:** Leland Ackerson, Elizabeth Procter-Gray, Qun Le, Kevin Kane, Wenjun Li

**Affiliations:** University of Massachusetts Lowell, Lowell, Massachusetts, United States; University of Massachusetts Lowell, Lowell, Massachusetts, United States; University of Massachusetts Lowell, Lowell, Massachusetts, United States; University of Massachusetts Lowell, Lowell, Massachusetts, United States; University of Massachusetts Lowell, Lowell, Massachusetts, United States

## Abstract

Neighborhood sociodemographic environmental factors may influence the risk of falling in older adults, which has important policy implications. We investigated the associations of neighborhood physical and social characteristics with rates of indoor and outdoor falls in a multi-racial cohort of 729 community-dwelling adults aged 65 to 99 residing in Massachusetts (2018-2024). Associations of Census tract-level sociodemographic and built environmental characteristics with fall rates were analyzed using negative binomial models. The overall rates of any, indoor and outdoor falls were 67.9 (95% CI: 63.9 – 72.2), 32.1 (95% CI: 29.4-35.1) and 35.6 (95% CI: 32.7-38.7) falls per 100 person-years, respectively. In crude analyses rates of any, indoor and outdoor falls were significantly associated with 12, 3 and 20 of the 80 neighborhood characteristics under analysis, respectively. After adjusting for individual age, sex, and race, 4, 7 and 6 remained statistically significant for any, indoor and outdoor falls, respectively (e.g., for outdoor falls % male, Hispanic population, % commuting 25-34 minutes, % of Black-occupied homes that are rented, % of Hispanic-occupied homes that are rented, and total number of Hispanic-occupied homes). After further adjustments for health-related risk factors, only a few neighborhood factors remained significant for either indoor or outdoor falls. In conclusion, neighborhood characteristics are more likely associated with outdoor falls than indoor falls. The observed associations likely result from compositional effects rather than contextual effects. When designing community-based falls prevention programs, the population compositional effects and geographic distribution of older adults with higher fall risks should be carefully considered.

